# γδ T cells in cancer immunotherapy

**DOI:** 10.18632/oncotarget.13051

**Published:** 2016-11-03

**Authors:** Chang Zou, Pan Zhao, Zhangang Xiao, Xianghua Han, Fan Fu, Li Fu

**Affiliations:** ^1^ Clinical Medical Research Center, The Second Clinical College of Jinan University, Shenzhen Peoples Hospital, Shenzhen, China; ^2^ Research Institute of Shenzhen Hornetcorn biotechnology, Shenzhen, China; ^3^ Department of Pharmacology, Laboratory of Molecular Pharmacology, School of Pharmacy, Southwest Medical University, Luzhou, Sichuan, PR China; ^4^ Department of Pharmacology, Shenzhen Key Laboratory of translational Medicine of Tumor and Cancer Research Centre, School of Medicine, Shenzhen University, Shenzhen, China

**Keywords:** γδ T cells, immunotherapy, anti-tumor, cancer treatment

## Abstract

γδ T cells are one of the three immune cell types that express antigen receptors. They contribute to lymphoid antitumor surveillance and bridge the gap between innate and adaptive immunity. γδ T cells have the capacity of secreting abundant cytokines and exerting potent cytotoxicity against a wide range of cancer cells. γδ T cells exhibit important roles in immune-surveillance and immune defense against tumors and have become attractive effector cells for cancer immunotherapy. γδ T cells mediate anti-tumor therapy mainly by secreting pro-apoptotic molecules and inflammatory cytokines, or through a TCR-dependent pathway. Recently, γδ T cells are making their way into clinical trials. Some clinical trials demonstrated that γδ T cell-based immunotherapy is well tolerated and efficient. Despite the advantages that could be exploited, there are obstacles have to be addressed for the development of γδ T cell immunotherapies. Future direction for immunotherapy using γδ T cells should focus on overcoming the side effects of γδ T cells and exploring better antigens that help stimulating γδ T cell expansion *in vitro*.

## INTRODUCTION

Immunotherapy is one of the attracted areas in developing novel anti-tumor therapeutics. The adoptive immunotherapy is accomplished by expanding immune effector cells *in vitro* and transferring the activated immune cells into the hosts, that target against tumor cells or stimulate immune response to eliminate tumor cells [1–3]. There are two main categories of T lymphocytes: αβ and γδ T cells. The difference of these two types of T cells is that they expressed different cell surface antigen receptors [4]. The majority of αβ T cells recognize antigenic peptides with major histocompatibility complex (MHC) class I or class II[5]. In the peripheral blood, αβ T cells account for about 95%, while γδ T cells contribute to only 5% of total CD3^+^ cells [6]. Cells with the αβ cell surface receptors generally express CD4 or CD8 lineage markers. Most of the αβ T cells belong to helper or cytotoxic/effector subsets [7, 8]. In contrast, γδ T cells do not usually express CD4 or CD8 lineage markers, and they do not require conventional MHC antigen presentation [6]. γδ T cells have the capacity of secreting abundant cytokines. They exert potent cytotoxicity against a wide range of malignancies [9–11]. Therefore, γδ T cells have become the attractive effector cells for cancer immunotherapy. This review will discuss the classification and characteristics of γδ T cells, the roles of,γδ T cells in anti-cancer therapy, the progress in clinical application using γδ T cells and the prospect of developmental direction of γδ T cells in the future.

## CLASSIFICATION OF γδ T CELLS

Human γδ T cells are subdivided into Vδ1, Vδ2 and Vδ3 T cells based on their surface antigen. They are a group of unconventional T cells [12]. Typically, about 50% to 75% of γδ T lymphocytes in peripheral-blood express Vδ2 chain, and co-express Vγ9 chain. These cells are named Vγ9Vδ2 T cells. Vγ9Vδ2 T cells present only in humans and nonhuman primates [13] and contribute to 1% to 10% of T cells in the peripheral blood of healthy human [14, 15]. Activated Vδ2 T cells express cell adhesion molecules, such as CD86, CD80 and MHC-II[16]. They show the characteristics of professional antigen presenting cells[16]. Vγ9Vδ2 T cells have the unique feature of recognizing non-peptidic phosphoantigens[17]. These cells proliferate vigorously *in vitro* in response to stimulation of microbial or synthetic phosphoantigens [6]. They play a critical role in anti-infection immunity and anti-tumor surveillance [18, 19]. Activated Vγ9Vδ2 T cells express granulysin, perforin, Fas/Fas ligand (FasL), granzyme-A and B,to kill the asexual stages of P.falciparum and inhibit the growth of intraerythrocytic stages of P.falciparum in the blood [20]. In addition, activated Vγ9Vδ2 T cells express TGF-β, IL-4 and IL-10. They also inhibit T cell proliferation [21].

The second subset of γδ T cells has the Vδ1 chain. Vδ1^+^ T cells are more prevalent in tissues than in the peripheral blood. Most of the tissue-associated γδ T cells possess the function of defending against epithelial cancers [22–24]. Vδ1 chain is prominent in the intraepithelial layer of mucosal surface [25]. Vδ1^+^ T cells protect epithelial tissue integrity against cell transformation, tissue damage or infection [26, 27].

Both Vδ1 and Vδ2 T cell subsets have almost equal amounts of NKG2D^+^ cells and CD6^+^ cells.. The Vδ1 subset has more IFN-γ-producing cells and CD27^+^CD45RA^-^ cells than the Vδ2 subset [22]. In addition, the peripheral Vδ2 can be expanded by phosphoantigens. The anti-γδ Ab is a potent stimulus that could expand both Vδ1 and Vδ2 subsets [22]. The anti-CD3 Ab [28, 29] or concanavalin A [22, 28] can also be used to expand both Vδ1 and Vδ2 subsets.

Besides Vδ1 and Vδ2 cells, there is a very small subset of Vδ3 T cells. Little is known about this human γδ T cells, except for the indirect evidence of their immunity against CMV and HIV [30–33]. Although there are only 0.2% of circulating T cells consist of Vδ3 T cells, Vδ3 T cells, are rich in liver and they are found in patients with leukemia and some chronic viral infections [34].

## THE ROLES OF γδ T CELLS IN IMMUNE RESPONSES

γδ T cells play various roles in immune response. They promote immune responses by interacting with other immune cells. They also secrete different cytokines, chemokines and growth factors. [33, 35]. Other important roles include recruiting macrophages, cytolytic activity et al [35].

Firstly, cytotoxicity is one of the important roles of γδ T cells. The cytotoxicity of Vγ9Vδ2 T-cell is accomplished by producing a variety of chemokines and cytokines, such as perforin-granzyme, tumor necrosis factor (TNF)/TNF receptor (TNF/TNFR) and TNF-related apoptosis-inducing ligand (TRAIL)/TRAIL receptor (TRAILR) systems [36,37]. In addition, γδ T cells proliferated in response to NK cell ligands and exhibited cytotoxicity against K562 cells [38]. Mattarollo and his colleagues demonstrated high levels of cytotoxicity against solid tumor-derived cells with Vγ9Vδ2 T cells, chemotherapeutic agents, bisphosphonate and zoledronate [36, 39]. Furthermore, γδ T cells regulate other immune and non-immune cells. The roles of γδ T cells as critical early responders and cytokine producers have been further demonstrated by Ferrick and colleagues [40]. Their studies showed that γδ T cells were the major initial producers of interferon (IFN)-γ after Listeria monocytogenes infection and IL-4 after Nippostrongylus brasiliensis infection. γδ T cells produce inflammatory cytokines that can directly attack infected cells and they establish a memory response to destroy pathogens upon re-exposure [41, 42]. The major cytokine produced by γδ T cells is IFN-γ which is a central cytokine in anti-tumor immune responses. IFN-γ plays an important role in antiviral, anti-bacterial and anti-tumor immunity [43–45].

**Figure 1:**
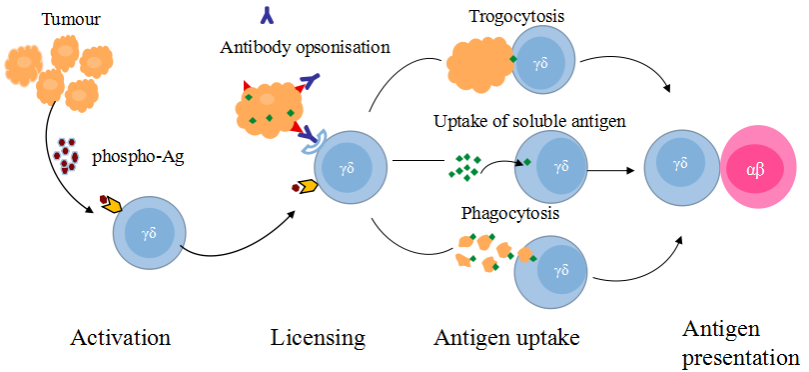
Ag presentation functions of γδ T cells At the tumor site, human γδ T cells have the potential to take up Ags directly by trogocytosis, phagocytosis or pinocytosis and cross-present the exogenous Ag to Ag-experimented and in-experimented αβ T cells.

Secondly, another important role of γδ T cells is antigen-presentation. Dendritic cells (DCs) are the most efficient antigen-presenting cells (APCs). They links the innate and adaptive immunity [30, 46]. To date, there are serious drawbacks in DCs-based adoptive immunotherapy. These include the limited expanding capacity and heterogenous function of DCs. These limitations should be overcome by searching for alternative sources of APCs [30, 47]. Activated γδ T cells have the functions and characteristics of professional APCs. They can also present specific antigens by MHC or MHC-related molecules [46]. In addition, activated γδ T cells have the capacity of inducing primary CD4+ and CD8+ T cell responses to antigens [12, 48–50]. Human γδ T cells have been characterized as professional APC because they were able to express MHC class II, co-stimulatory molecules and lymph node-homing chemokine receptors (e.g.CCL7)[46,51]. Yoshikawa found γδ T cells acquire antigen-presenting properties with CD86 expression by studying the function of activated γδ T cells with co-cultures in the absence of zoledronate [12]. In the presence of antibody-opsonized target cells, γδ T cells display both innate cytotoxic function and antigen-presenting capability. They share the characteristics of both the innate and adaptive immunity, [46,52]. Phosphoantigen-activated γδ T cells induce primary immune response in naive Ag-specific ɑ β T cells when given appropriate stimulation (Figure 1).

Thirdly, γδ T cells regulate immune responses by interacting with other cells. They assist B helper cells for generating IgA, IgM and IgG antibodies and play a regulatory role in humoral immunity [33, 53]. The interaction between γδ T cell and B cell is beneficial to immune responses in some species [54]. *In vitro*, activated Vγ9Vδ2 T cells provide B helper cell with CD40L, IL-10 and IL-4 [52, 53]. In addition, γδ T cells can activate immature DCs. Immature DCs co-cultured with phosphoantigens stimulated γδ T cells leads to a significantly increased expression of CD86 and MHC class I molecules, as well as acquiring the functional characteristics of activated DCs [4,55]. Moreover, γδ T cells induce DCs maturation by TCR-CD1 [39, 57] and Fas-FasL interaction [33, 58]. In addition, Vγ9Vδ2 T cells engage transformed cells through a series of innate receptor system, such as NKG2D [61]. NKG2D is a lectin C receptor and plays important function in ligand recognition by γδ T cells. NKG2D is expressed in many normal tissues and is overexpressed in most cancer-cells. It is required for tumor cell-recognition by Vγ9Vδ2 T cells. The key molecular determinants for tumor recognition by Vγ9Vδ2is a C-type lectin receptor and plays an important role in the ligand recognition by γδ T cells.T-cells come from NKG2D , which provides activation signals by binding to its ligands such as MIC and ULBP familiesis a C-type lectin receptor and plays an important role in the ligand recognition by γδ T cells.[62, 63]. On the other hand, human DCs can induce γδ T cell proliferation and mediate γδ T cell activation. This finding was confirmed by Ye et al [56].

## THE ROLES OF γδ T CELLS AGAINST MALIGNANCIES

**Figure 2:**
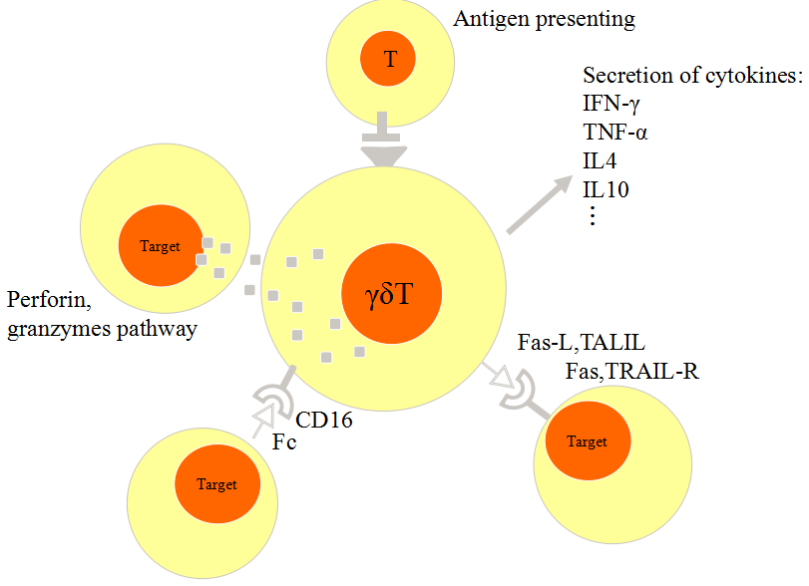
Schematic figure of anti-tumor activity of γδ T cells 1) γδ T cells secret IFN-γ and TNF-α, IL-4 and IL-10. 2) enhanced expression of Fas-L and TRAIL in γδ T cells. 3) γδ T cells express CD16 mediates Fc antibody-dependent cellular cytotoxicity (ADCC). 4) γδ T cells release perforin and granzymes for cytotoxic activity.

γδ T cells can directly identify malignant cells and reject them using body immunity. It is generally accepted that γδ T cells reject tumor cells mainly through the following ways. Firstly, cytokines mediate the lethal effect. γδ T cells exert the anti-tumor activity by generating various chemokines and cytokines, such as TNF-α and IFN-γ [34, 64]. IFN-γ can directly inhibit tumor growth, stimulate macrophages, and block angiogenesis [33]. In addition, γδ T cells secrete Th2-like cytokines such as IL-4 [65] and IL-10 [66], control CD8^+^ T cell expansion, adjust the expansion and recruitment of monocytes and neutrophils. Secondly, γδ T cells upregulate the expression of Fas ligand (Fas-L) and TNF-related apoptosis-inducing ligand (TRAIL) therefore enhance the tumor killing activity in the Fas-or TRAIL-receptor (R) sensitive tumors [63, 67–69]. Thirdly, some γδ T cells express CD16, which is a receptor for the Fc portion of immunoglobulin G (Fcγ receptors). CD16 can enhance the antibody-dependent cellular cytotoxicity (ADCC) in the presence of anti-tumor cell monoclonal antibodies[70, 71]. Fourthly, following T cell receptor-dependent activation, γδ T cells release granzymes and perforin that mediate cellular apoptosis. by activating related enzymes [72]. Finally, γδ T cells interacting with professional APCs, that process and display antigens and provide stimulated signals necessary for inducing the target cell killing [73] (Figure 2).

## IMMUNOTHERAPY USING ACTIVATED γδ T CELLS EX VIVO

γδ T cells possess unrestricted major histocompatibility complex (MHC) lytic activity and can react to bacteria, viruses, and tumors [32]. Therefore, they have become attractive target cells for adoptive cell transfer therapy [23, 30, 74]. iIn humans, this activity was exhibited *in vitro* against various tumors, including prostate cancer cells [75, 76]. Currently, one of the focus of the recent translational studies is the adoptive transfer of *ex vivo* activated and expanded γδ T cells [4]. Vγ9Vδ2 T cells dramatically expand following infection with prokaryotic or parasite pathogens [77, 78]. Bouet-Toussaint investigated their anti-tumor cytotoxicity by the means of *ex vivo* expansion to be utilized for adoptive immunotherapy. As a result, Vγ9Vδ2 T cells showed a strong lytic activity toward a wide spectrum of tumor cell lines including those from hepatocellular carcinomas and colorectal cancer, demonstrating a potential treatment application for these cancers [67]. Van Acker's study explains the activation of DC-mediated γδ T cellsactivation, including the cell-to-cell interaction mechanism, and forecasts its potential therapeutic use in DC-based cancer immunotherapy [4]. In a trial of adoptive transfer of expanded γδ T cells *in vitro* to non-small cell lung cancer patients, immune monitoring data showed that the number of peripheral γδ T cells gradually increased with increasing numbers of infusions [28, 79].

## CLINICAL APPLICATION OF γδ T CELLS

Targeting the immune system against tumors is a therapeutic measure although progress has been slow and success is limited. γδ T cells regulate anti-tumor reactions mainly by producing pro-apoptotic molecules and inflammatory cytokines, or through a TCR-dependent pathway [59]. The main obstacle of utilizing γδ T cells in anti-tumor therapy is that only a small quantity of γδ T cells is amplified without a continuous and reliable method in current clinical trials. Several studies have reported that the most efficient and widely used method to amplify γδ T cells is to utilize a phosphorylated antigen or anti-γδ TCR antibody [59, 80]. Because of their unrestricted MHC lytic activity *in vitro*, γδ T cells can be applied to anti-tumor therapies across the board [81]. In several clinical trials, γδ T cells been able topenetrate display efficacy in diverse tumors, including renal cell carcinomas [82], lung carcinomas [83], melanomas [84], breast cancer [85] and many others. It is also distinctive that γδ T cells are activated in response to tumors but not to normal cells *in vitro*. Furthermore, in clinical settings, γδ T cells were efficiently activated by phosphoantigens or bisphosphonates. Other compounds, such as zoledronate, pamidronate and alkylamine can indirectly activate γδ T lymphocytes [86–88]. This further bolsters the antineoplastic functions of this cell population *in vivo* for the treatment of cancers in human [85].

Recent clinical trials show that γδ T cells are becoming more apparent in therapeutic settings. For example, γδ T cells exhibit an effective role in genitourinary system tumor, and some trials have demonstrated the safety and efficient use of γδ T cell-based immunotherapy. Bennouna and his colleagues have reported a stable disease (SD) in six patients (60%) with metastatic renal cell carcinoma who underwent γδ T cells therapy [80]. In the study by Kobayashi and his colleagues, observedone In this study, they used low dose IL-2 *in vitro* to activate and expand the γδ T cells. This was met with a clinical response in which there was an incremental rise in the number of IFN-γ-producing adoptive transferred Vγ9Vδ2 T lymphocytes. They observed a complete remission in a patient with advanced renal cell carcinomas who underwent six monthly cycles of autologous γδ T cell therapy. The patient with complete remission was disease free for more than 3 years without any additional treatment [81]. The authors further expanded the research to a phase I/II clinical trial, in which 2-methyl-3-butenyl-1-pyrophosphate (2M3B1PP) combined with zoledronate and IL-2 were administered to patients with advanced renal cell carcinoma. The results showed prolonged tumor doubling time in all patients with 1 CR, 5 SD, and 5 Progressive Disease (PD). Objective clinical responses were achieved and the treatment regimen was well tolerated in patients with advanced renal cell carcinoma [82].

These studies in mice and in humans have provided rational for exploiting the potential application of usingγδ T cells in cancer immunotherapy. The main feature that makeγδ T cells attractive is their capacity to be easily and specifically stimulated either by phosphoantigens, such as HMBPP, and IPP , or by agents that induce IPP accumulation [61, 83, 84]. It is important to utilize the capacity of γδ T cells in cancer immunotherapy. Francesco Dieli and his colleagues have studied the *in vivo* function of Vγ9Vδ2 T cells activated by zoledronate and low-dose IL-2 and found that activated Vγ9Vδ2 T cells significantly inhibited the progression of the hormone-refractory prostate cancer.. The results show favorable clinical responses in six out of nine patients treated with zoledronate and IL-2 [86]. Table 1 summarized some important clinical trials using γδ T cells immunotherapy.

**Table 1:** γδ T cells anti-tumor effect in clinical studies

Cancer type	Immunotherapeutic approach	Results	References
Advanced renal carcinoma	Adoptive immunotherapy	3/5Prolongation of tumor DT	Kobayashi [74] et al 2007
Renal Cell Carcinoma with pulmonary metastasis	Adoptive immunotherapy	CR	Kobayashi [81] et al 2010
Advanced renal carcinoma	Adoptive immunotherapy	1/11 CR, 5/11 SD	Kobayashi [82] et al 2011
Metastatic renal carcinoma	In vivo Zoledronate and IL-2	3 PR, 5 SD	Dieli [86] et al 2007
Renal cell carcinoma	Adoptive immunotherapy	6/10SD	Bennouna [80] et al 2008
Renal cell carcinoma	Adoptive immunotherapy	2/12SD	Lang [87] et al 2011
Metastatic renal carcinoma	Adoptive immunotherapy	Causing target cell dissolution and death	Viey [77] et al 2005
Multiple myeloma	Adoptive immunotherapy	4/6SD	Abe [88] et al 2009
Myeloma carcinoma	Adoptive immunotherapy	PR	Knight [76] et al 2012
Advanced lung cancer	Adoptive immunotherapy	6/10SD	Nakajima [89] at al 2010
Melanoma	Adoptive immunotherapy	Causing target cell dissolution and death	Cordova [72] et al 2012
Malignant leukemia	In vivo Zoledronate and IL-2	3/4CR	Wilhelm [90] et al 2014

Abbreviations: Stable Disease (SD), Partial Response (PR), Complete Remission (CR), Doubling Time (DT)

## CONCLUSION AND FUTURE PERSPECTIVE

Recent advances in tumor immunology have confirmed the crucial roles of immune suppressive cells and immune checkpoint systems in inhibiting tumor immune responses in cancer patients. We have reviewed the features and clinical trials of immunotherapies using γδ T cells conducted in the past years. Multiple trials proved that immunotherapies using γδ T cells were safe and well tolerated. Although γδ T cell-based immunotherapies have advantages that worth exploited, some obstacles have to be overcome. There are some aspects that could be improved in future clinical trials. Firstly, it has been revealed that repeated application of phosphoantigens might lead to inability, exhaustion or even death of effector cells. Efforts should be focus on overcoming the anergy effect and extending the functions of γδ T cells. This could be achieved by efficiently expanding Vγ9Vδ2 T cells with Zol plus IL-2 *in vitro*. Secondly, immunotherapy may induce significant adverse reactions. Activated γδ T cells can produce proinflammatory cytokines that may elicit severe adverse reactions. The clinical efficacy of γδ T cell immunotherapy should be further assessed. Combinations of newly emerging therapy with established treatments could minimize the potential side effects of immune reconstitution in the future. The risk for severe adverse reactions and anergy should be evaluated by controlled human clinical trials. Thirdly, better antigens should be sought to help stimulating γδ T cell *in vitro* so that a large amount of cells could be prepared for adoptive cell transfer. Finally, it is needed to overcome the major challenge with combined tumor-targeting antibodies.. γδ T cells and tumor-targeting antibodies might become significant modality for cancer immunotherapy in future.

## References

[1] Silva-Santos B., Serre K., Norell H (2015). γδ T cells in cancer. Nature Reviews Immunology.

[2] Hu Z, Xia J, Fan W, Wargo J, Yang YG (2016). Human melanoma immunotherapy using tumor antigen-specific T cells generated in humanized mice. Oncotarget.

[3] Paret C, Simon P, Vormbrock K, Bender C, Kölsch A, Breitkreuz A, Ö Yildiz, Omokoko T, Hubich-Rau S, Hartmann C, Häcker S, Wagner M, Roldan DB (2015). CXorf61 is a target for T cell based immunotherapy of triple-negative breast cancer. Oncotarget.

[4] Van Acker H.H., Sebastien Anguille, Viggo F Van Tendeloo, Eva Lion. (2015). Empowering gamma delta T cells with antitumor immunity by dendritic cell-based immunotherapy. Oncolimmunology.

[5] Adams E.J., Havran W.L (2015). Introduction to Cellular Immunology Special Issue on γδ T cells. Cellular Immunology.

[6] Tanaka Y, Morita C T, Nieves E, Brenner M B, Bloom B R (1995). Natual and synthetic non-peptide antigens recognized by human γδ T cells. Letter to nature.

[7] Knies D, Klobuch S, Xue SA, Birtel M, Echchannaoui H, Yildiz O, Omokoko T, Guillaume P, Romero P, Stauss H, Sahin U, Herr W, Theobald M (2016). An optimized single chain TCR scaffold relying on the assembly with the native CD3-complex prevents residual mispairing with endogenous TCRs in human T-cells. Oncotarget.

[8] Blaeschke F, Thiel U, Kirschner A, Thiede M, Rubio RA, Schirmer D, Kirchner T, Richter GH, Mall S, Klar R, Riddell S, Busch DH, Krackhardt A (2016). Human HLA-A*02: 01/CHM1+ allo-restricted T cell receptor transgenic CD8+ T Cells specifically inhibit Ewing sarcoma growth in vitro and in vivo. Oncotarget.

[9] Tyler CJ, Doherty DG, Moser B, Eberl M (2015). Human Vγ9/Vδ2 T cells: Innate adaptors of the immune system. Cellular Immunology.

[10] Rosmalen J.G., van W (2002). Ewijk and P.J.Leenen. T-cell education in autoimmune diabetes: teachers and students. Trends Immunol.

[11] Braza M.S., Klein B (2013). Anti-tumour immunotherapy with Vγ9Vδ2 T lymphocytes: from the bench to the bedside. British Journal of Haematology.

[12] Yoshikawa T, Takahara M, Tomiyama M, Nieda M, Maekawa R, Nakatsura T (2014). Large-scale expansion of gammadelta T cells and peptide-specific cytotoxic T cells using zoledronate for adoptive immunotherapy. Int J Oncol.

[13] Rakasz E, MacDougall AV, Zayas MT, Helgelund JL, Ruckward TJ, Hatfield G, Dykhuizen M, Mitchen JL, Evans PS, Pauza CD (2000). Gammadelta T cell receptor repertoire in blood and colonic mucosa of rhesus macaques. J Med Primatol.

[14] Adams E.J., Gu S., Luoma A.M (2015). Human gamma delta T cells: Evolution and ligand recognition. Cell Immunol.

[15] Morita C.T., Mariuzza R.A., Brenner M.B (2000). Antigen recognition by human gamma delta T cells: pattern recognition by the adaptive immune system. Springer Semin Immunopathol.

[16] Li H., Pauza C.D (2011). Rapamycin increases the yield and effector function of human gammadelta T cells stimulated in vitro. Cancer Immunol Immunother.

[17] Lafont V, Sanchez F, Laprevotte E, Michaud HA, Gros L, Eliaou JF, Bonnefoy N (2014). Plasticity of gammadelta T Cells: Impact on the Anti-Tumor Response. Front Immunol.

[18] Bonneville M., Scotet E (2006). Human Vgamma9Vdelta2 T cells: promising new leads for immunotherapy of infections and tumors. Curr Opin Immunol.

[19] Riganti C, Massaia M, Davey MS, Eberl M (2012). Human gammadelta T-cell responses in infection and immunotherapy: common mechanisms, common mediators?. Eur J Immunol.

[20] Farouk SE, Mincheva-Nilsson L, Krensky AM, Dieli F, Troye-Blomberg M (2004). Gamma delta T cells inhibit in vitro growth of the asexual blood stages of Plasmodium falciparum by a granule exocytosis-dependent cytotoxic pathway that requires granulysin. Eur J Immunol.

[21] Kühl AA, Pawlowski NN, Grollich K, Blessenohl M, Westermann J, Zeitz M, Loddenkemper C, Hoffmann JC (2009). Human peripheral gammadelta T cells possess regulatory potential. Immunology.

[22] Zhou J, Kang N, Cui L, Ba D, He W (2012). Anti-gammadelta TCR antibody-expanded gammadelta T cells: a better choice for the adoptive immunotherapy of lymphoid malignancies. Cell Mol Immunol.

[23] Qi J, Zhang J, Zhang S, Cui L, He W (2003). Immobilized MICA could expand human Vdelta1 gammadelta T cells in vitro that displayed major histocompatibility complex class I chain-related A-dependent cytotoxicity to human epithelial carcinomas. Scand J Immunol.

[24] Kabelitz D., Wesch D.W. (2007). He.Perspectives of gammadelta T cells in tumor immunology. Cancer Res.

[25] Kabelitz D., Glatzel A., Wesch D (2000). Antigen recognition by human gammadelta T lymphocytes. Int Arch Allergy Immunol.

[26] Gould D.S., Ploegh H.L., Schust D.J (2001). Murine female reproductive tract intraepithelial lymphocytes display selection characteristics distinct from both peripheral and other mucosal T cells. J Reprod Immunol.

[27] Poles MA, Barsoum S, Yu W, Yu J, Sun P, Daly J, He T, Mehandru S, Talal A, Markowitz M, Hurley A, Ho D, Zhang L (2003). Human immunodeficiency virus type 1 induces persistent changes in mucosal and blood gammadelta T cells despite suppressive therapy. J Virol.

[28] Siegers GM, Dhamko H, Wang XH, Mathieson AM, Kosaka Y, Felizardo TC, Medin JA, Tohda S, Schueler J, Fisch P, Keating A (2011). Human Vdelta1 gammadelta T cells expanded from peripheral blood exhibit specific cytotoxicity against B-cell chronic lymphocytic leukemia-derived cells. Cytotherapy.

[29] Dokouhaki P, Han M, Joe B, Li M, Johnston MR, Tsao MS, Zhang L (2010). Adoptive immunotherapy of cancer using ex vivo expanded human gammadelta T cells: A new approach. Cancer Lett.

[30] Deniger D.C., Moyes J.S., Cooper L.J (2014). Clinical applications of gamma delta T cells with multivalent immunity. Front Immunol.

[31] Knight A, Madrigal AJ, Grace S, Sivakumaran J, Kottaridis P, Mackinnon S, Travers PJ, Lowdell MW (2010). The role of Vdelta2-negative gammadelta T cells during cytomegalovirus reactivation in recipients of allogeneic stem cell transplantation. Blood.

[32] Kabelitz D, Hinz T, Dobmeyer T, Mentzel U, Marx S, Böhme A, Arden B, Rossol R, Hoelzer D (1997). Clonal expansion of Vgamma3/Vdelta3-expressing gammadelta T cells in an HIV-1/2-negative patient with CD4 T-cell deficiency. Br J Haematol.

[33] Wu YL, Ding YP, Tanaka Y, Shen LW, Wei CH, Minato N, Zhang W (2014). gammadelta T cells and their potential for immunotherapy. Int J Biol Sci.

[34] S TodroM Meraviglia, Caccamo N, Stassi G Dieli F (2013). Combining conventional chemotherapy and gammadelta T cell-based immunotherapy to target cancer-initiating cells. Oncoimmunology.

[35] Rei M., Pennington D.J., Silva-Santos B (2015). The emerging Protumor role of gammadelta T lymphocytes: implications for cancer immunotherapy. Cancer Res.

[36] Mattarollo SR, Kenna T, Nieda M, Nicol AJ (2007). Chemotherapy and zoledronate sensitize solid tumour cells to Vgamma9Vdelta2 T cell cytotoxicity. Cancer Immunol Immunother.

[37] Bonneville M, Chen ZW, Déchanet-Merville J, Eberl M, Fournié JJ, Jameson JM, Lopez RD (2015). Massai Chicago 2014--30 years of gammadelta T cells. Cell Immunol.

[38] Shimizu K, Shinga J, Yamasaki S, Kawamura M, Dörrie J, Schaft N, Sato Y, Iyoda T, Fujii S (2015). Transfer of mRNA Encoding Invariant NKT Cell Receptors Imparts Glycolipid Specific Responses to T Cells and gammadeltaT Cells. PLoS One.

[39] Zheng J, Liu Y, Lau YL, Tu W (2013). gammadelta-T cells: an unpolished sword in human anti-infection immunity. Cell Mol Immunol.

[40] Carding S.R., Egan P.J (2002). Gammadelta T cells: functional plasticity and heterogeneity. Nat Rev Immunol.

[41] García VE, Sieling PA, Gong J, Barnes PF, Uyemura K, Tanaka Y, Bloom BR, Morita CT, Modlin RL (1997). Single-cell cytokine analysis of gamma delta T cell responses to nonpeptide mycobacterial antigens. J Immunol.

[42] Chen M, Hu P, Ling N, Peng H, Lei Y, Hu H, Zhang D, Ren H (2015). Enhanced functions of peripheral gammadelta T cells in chronic hepatitis B infection during interferon alpha treatment in vivo and in vitro. PLoS One.

[43] Cimini E, Bonnafous C, Bordoni V, Lalle E, Sicard H, Sacchi A, Berno G, Gioia C, D’Offizi G (2012). Visco Interferon-alpha improves phosphoantigen-induced Vgamma9Vdelta2 T-cells interferon-gamma production during chronic HCV infection. PLoS One.

[44] Vantourout P., Hayday A (2013). Six-of-the-best: unique contributions of gammadelta T cells to immunology. Nat Rev Immunol.

[45] Tyler CJ, Doherty DG, Moser B, Eberl M (2015). Human Vgamma9/Vdelta2 T cells: Innate adaptors of the immune system. Cell Immunol.

[46] Brandes M., Willimann K., Moser B (2005). Professional antigen-presentation function by human gammadelta T Cells. Science.

[47] Moser B., Eberl M (2011). Gammadelta T-APCs: a novel tool for immunotherapy?. Cell Mol Life Sci.

[48] Landmeier S, Altvater B, Pscherer S, Juergens H, Varnholt L, Hansmeier A, Bollard CM, Moosmann AB. (2009). Activated human gammadelta T cells as stimulators of specific CD8+ T-cell responses to subdominant Epstein Barr virus epitopes: potential for immunotherapy of cancer. J Immunother.

[49] Altvater B, Pscherer S, Landmeier S, Kailayangiri S, Savoldo B, Juergens H, Rossig C (2012). Activated human gammadelta T cells induce peptide-specific CD8+ T-cell responses to tumor-associated self-antigens. Cancer Immunol Immunother.

[50] Adams E.J., Havran W.L (2015). Introduction to Cellular Immunology Special Issue on gammadelta T cells. Cell Immunol.

[51] Brandes M., Willimann K., Bioley G., Le ´vy N., Eberl M., Luo M., Tampe R, Le ´vy F., Romero P.B. Moser.Cross-presenting human gammadelta T cells induce robust CD8+ alphabeta T cell responses. Proc. Natl. Acad.

[52] Himoudi N, Morgenstern DA, Yan M, Vernay B Saraiva L, Wu Y, Cohen CJ, Gustafsson K, Anderson J (2012). Human γδ T lymphocytes are licensed for professional antigen presentation by interaction with opsonized target cells. J Immunol.

[53] Caccamo N, Battistini L, Bonneville M, Poccia F, Fournié JJ, Meraviglia S, Borsellino G, Kroczek RA (2006). CXCR5 identifies a subset of Vgamma9Vdelta2 T cells which secrete IL-4 and IL-10 and help B cells for antibody production. J Immunol.

[54] Anderson J, Gustafsson K, Himoudi N, Yan M, Heuijerjans J (2012). Licensing of gammadelta T cells for professional antigen presentation: A new role for antibodies in regulation of antitumor immune responses. Oncoimmunology.

[55] E Lo Presti, Caccamo N, Orlando V, Dieli F, Meraviglia S (2016). Activation and selective IL-17 response of human Vγ9Vδ2 T lymphocytes by TLR-activated plasmacytoid dendritic cell. Oncotarget.

[56] Ye Z, Haley S, Gee AP, Henslee-Downey PJ, Lamb LS (2002). In vitro interactions between gamma deltaT cells,DC, and CD4+ T cells; implications for the immunotherapy of leukemia. Cytotherapy.

[57] Hayday A.C (2000). [gamma][delta] cells: a right time and a right place for a conserved third way of protection. Annu Rev Immunol.

[58] Tyler CJ, Doherty DG, Moser B, Eberl M (2015). Human Vgamma9/Vdelta2 T cells: Innate adaptors of the immune system. Cell Immunol.

[59] Cabillic F, Toutirais O, Lavoué V, CT de La Pintière, Daniel P, Rioux-Leclerc N, Turlin B, Mönkkönen H, Mönkkönen J, Boudjema K, Catros V, Bouet-Toussaint F (2010). Aminobisphosphonate-pretreated dendritic cells trigger successful Vγ9Vδ2 T cell amplification for immunotherapy in advanced cancer patients. Cancer Immunology, Immunotherapy.

[60] Castella B, Riganti C, Fiore F, Pantaleoni F, Canepari ME, Peola S, Foglietta M, Palumbo A, Bosia A, Coscia M (2011). Immune modulation by zoledronic acid in human myeloma: an advantageous cross-talk between Vgamma9Vdelta2 T cells, alphabeta CD8C T cells, regulatory T cells, and dendritic cells. J Immunol.

[61] Dieli F, Vermijlen D, Fulfaro F, Caccamo N, Meraviglia S, Cicero G, Roberts A, Buccheri S, D’Asaro M, Gebbia N, Salerno A, Eberl M, Hayday AC (2007). Targeting human {gamma}delta} T cells with zoledronate and interleukin-2 for immunotherapy of hormone-refractory prostate cancer. Cancer Res.

[62] T1 Lança, Correia DV, Moita CF, Raquel H, Neves-Costa A, Ferreira C, Ramalho JS, Barata JT, Moita LF, Gomes AQ, Silva-Santos B (2010). The MHC class Ib protein ULBP1 is a nonredundant determinant of leukemia/lymphoma susceptibility to gammadelta T-cell cytotoxicity. Blood.

[63] Kong Y, Cao W, Xi X, Ma C, Cui L, He W (2009). The NKG2D ligand ULBP4 binds to TCR gamma9/delta2 and induces cytotoxicity to tumor cells through both TCR gammadelta and NKG2D. Blood.

[64] Liu Z, Guo BL, Gehrs BC, Nan L, Lopez RD (2005). ex vivo expanded human Vgamma9Vdelta2+ gammadelta-T cells mediate innate antitumor activity against human prostate cancer cells in vitro. J Urol.

[65] Beetz S, Wesch D, Marischen L (2008). Innate immune functions of human gammadelta T cells. Immunobiology.

[66] Rhodes KA, Andrew EM, Newton DJ, Tramonti D, Carding SR (2008). A subset of IL-10-producing γδ T cells protect the liver from Listeria-elicited, CD8+T cell-mediated injury.European. Journal of Immunology.

[67] Bouet-Toussaint F, Cabillic F, Toutirais O, Le Gallo M, Thomas de la Pintière C, Daniel P, Genetet N, Meunier B, Dupont-Bierre E, Boudjema K, Catros V (2008). Vgamma9Vdelta2 T cell-mediated recognition of human solid tumors. Potential for immunotherapy of hepatocellular and colorectal carcinomas. Cancer Immunol Immunother.

[68] Zheng BJ, Ng SP, Chua DT, Sham JS, Kwong DL, Lam CK, Ng MH (2002). Peripheral gamma delta T-cell deficit in nasopharyngeal carcinoma. Int J Cancer.

[69] Kondo M, Sakuta K, Noguchi A, Ariyoshi N, Sato K, Sato S, Sato K, Hosoi A, Nakajima J, Yoshida YS. (2008). Zoledronate facilitates large-scale ex vivo expansion of functional γδ T cells from cancer patients for use in adoptive immunotherapy. Cytotherapy.

[70] Sakamoto M, Nakajima J, Murakawa T, Fukami T, Yoshida Y, Murayama T, Takamoto S, Matsushita H, Kakimi K (2011). Adoptive immunotherapy for advanced non-small cell lung cancer using zoledronate-expanded gammadeltaTcells: a phase I clinical study. J Immunother.

[71] Sugie T, Murata-Hirai K, Iwasaki M, Morita CT, Li W, Okamura H, Minato N, Toi M, Tanaka Y (2013). Zoledronic acid-induced expansion of γδ T cells from early-stage breast cancer patients: effect of IL-18 on helper NK cells. Cancer Immunology, Immunotherapy.

[72] Cordova A, Toia F, C La Mendola, Orlando V, Meraviglia S, Rinaldi G, Todaro M., Cicero G, Zichichi L, Donni PL, Caccamo N, Stassi G, Dieli F (2012). Characterization of human gammadelta T lymphocytes infiltrating primary malignant melanomas. PLoS One.

[73] Zgani I, Menut C, Seman M, Gallois V, Laffont V, Liautard J, Liautard JP, Criton M, Montero JL (2004). Synthesis of Prenyl Pyrophosphonates as New Potent Phosphoantigens Inducing Selective Activation of Human Vγ9Vδ2 T Lymphocytes. Journal of Medicinal Chemistry.

[74] Kobayashi H, Tanaka Y, Yagi J, Osaka Y, Nakazawa H, Uchiyama T, Minato N, Toma H (2007). Safety profile and anti-tumor effects of adoptive immunotherapy using gamma-delta T cells against advanced renal cell carcinoma: a pilot study. Cancer Immunology, Immunotherapy.

[75] Kazuhiro Kakimi Hirokazu Matsushita Tomohiro Murakawa Nakajima J. (2014). T cell therapy for the treatment of non-small cell lung cancer[J].Transl. Lung Cancer Res.

[76] Knight A., Mackinnon S., Lowdell M.W (2012). Human Vdelta1 gamma-delta T cells exert potent specific cytotoxicity against primary multiple myeloma cells. Cytotherapy.

[77] Viey E, Fromont G, Escudier B, Morel Y, S Da Rocha, Chouaib S, Caignard A (2005). Phosphostim-Activated T Cells Kill Autologous Metastatic Renal Cell Carcinoma. The Journal of Immunology.

[78] Thompson Keith, Rojas-Navea Javier, Michael J (2006). Rogers.Alkylamines cause Vgamma 9Vdelta 2 T-cell activation and proliferation by inhibiting the mevalonate pathway. Blood.

[79] Roelofs AJ, Jauhiainen M, Mönkkönen H, Rogers MJ, Mönkkönen J, Thompson K (2009). Peripheral blood monocytes are responsible for γδ T cell activation induced by zoledronic acid through accumulation of IPP/DMAPP. British Journal of Haematology.

[80] Bennouna J, Bompas E, Neidhardt EM, Rolland F, Philip I, Galéa C, Salot S, Saiagh S, Audrain M, Rimbert M, Lafaye-de Micheaux S, Tiollier J, Négrier S (2008). Phase-I study of Innacell γδ™, an autologous cell-therapy product highly enriched in γ9δ2 T lymphocytes, in combination with IL-2, in patients with metastatic renal cell carcinoma. Cancer Immunology, Immunotherapy.

[81] Kobayashi H, Tanaka Y, Shimmura H, Minato N, Tanabe K (2010). Complete remission of lung metastasis following adoptive immunotherapy using activated autologous gammadelta T-cells in a patient with renal cell carcinoma. Anticancer Res.

[82] Kobayashi H, Tanaka Y, Yagi J, Minato N, Tanabe K (2011). Phase I / II study of adoptive transfer of γδ T cells in combination with zoledronic acid and IL-2 to patients with advanced renal cell carcinoma. Cancer Immunology, Immunotherapy.

[83] Johnson JR, Williams G, Pazdur R End Points and United States Food and Drug Administration Approval of Oncology Drugs.Journal of. Clinical Oncology.

[84] Body JJ (2006). Bisphosphonates for malignancy-related bone disease: current status, future developments. Supportive Care in Cancer.

[85] Jemal A, Murray T, Ward E, Samuels A, Tiwari RC, Ghafoor A, Feuer EJ Thun MJ .Cancer Statistics, American Cancer Society.

[86] Dieli F, Vermijlen D, Fulfaro F, Caccamo N, Meraviglia S, Cicero G, Roberts A, Buccheri S (2007). D’Asaro. Targeting Human T Cells with Zoledronate and Interleukin-2 for Immunotherapy of Hormone-Refractory Prostate Cancer. Cancer Research.

[87] Lang JM, Kaikobad MR, Wallace M, Staab MJ, Horvath DL, Wilding G, Liu G, Eickhoff JC, McNeel DG (2011). Malkovsky. Pilot trial of interleukin-2 and zoledronic acid to augment γδ T cells as treatment for patients with refractory renal cell carcinoma. Cancer Immunology, Immunotherapy.

[88] Abe Y, Muto M, Nieda M, Nakagawa Y, Nicol A, Kaneko T, Goto S, Yokokawa K, Suzuki K (2009). Clinical and immunological evaluation of zoledronate-activated Vγ9γδ T-cell-based immunotherapy for patients with multiple myeloma. Experimental Hematology.

[89] .Nakajima J, Murakawa T, Fukami T, Goto S, Kaneko T, Yoshida Y, Takamoto S, Kakimi K (2010). A phase I study of adoptive immunotherapy for recurrent non-small-cell lung cancer patients with autologous γδ T cells. European Journal of Cardio-Thoracic Surgery.

[90] Wilhelm M, Smetak M, Schaefer-Eckart K, Kimmel B, Birkmann J, Einsele H, Kunzmann V Successful adoptive transfer and in vivo expansion of haploidentical γ δ T cells.Journal of Translational Medicine.

